# Integrating Health Systems and Science to Respond to COVID-19 in a Model District of Rural Madagascar

**DOI:** 10.3389/fpubh.2021.654299

**Published:** 2021-07-21

**Authors:** Rado J. L. Rakotonanahary, Herinjaka Andriambolamanana, Benedicte Razafinjato, Estelle M. Raza-Fanomezanjanahary, Vero Ramanandraitsiory, Fiainamirindra Ralaivavikoa, Andritiana Tsirinomen'ny Aina, Lea Rahajatiana, Luc Rakotonirina, Justin Haruna, Laura F. Cordier, Megan B. Murray, Giovanna Cowley, Demetrice Jordan, Mark A. Krasnow, Patricia C. Wright, Thomas R. Gillespie, Michael Docherty, Tara Loyd, Michelle V. Evans, John M. Drake, Calistus N. Ngonghala, Michael L. Rich, Stephen J. Popper, Ann C. Miller, Felana A. Ihantamalala, Andriamihaja Randrianambinina, Bruno Ramiandrisoa, Emmanuel Rakotozafy, Albert Rasolofomanana, Germain Rakotozafy, Manuela C. Andriamahatana Vololoniaina, Benjamin Andriamihaja, Andres Garchitorena, Julio Rakotonirina, Alishya Mayfield, Karen E. Finnegan, Matthew H. Bonds

**Affiliations:** ^1^PIVOT NGO, Ranomafana, Madagascar; ^2^Madagascar Ministry of Public Health, Antananarivo, Madagascar; ^3^Department of Global Health and Social Medicine, Harvard Medical School, Boston, MA, United States; ^4^Centre Valbio, Ranomafana, Madagascar; ^5^Department of Biochemistry, Stanford University, Stanford, CA, United States; ^6^Institute for the Conservation of Tropical Environments, Stony Brook University, Stony Brook, NY, United States; ^7^Department of Anthropology, Stony Brook University, Stony Brook, NY, United States; ^8^Department of Environmental Sciences and Program in Population Biology, Ecology, and Evolutionary Biology, Emory University, Atlanta, GA, United States; ^9^Department of Environmental Health, Rollins School of Public Health, Emory University, Atlanta, GA, United States; ^10^Odum School of Ecology and Center for the Ecology of Infectious Diseases, University of Georgia, Athens, GA, United States; ^11^Department of Mathematics, University of Florida, Gainesville, FL, United States; ^12^Emerging Pathogens Institute, University of Florida, Gainesville, FL, United States; ^13^Center for African Studies, University of Florida, Gainesville, FL, United States; ^14^Brigham and Women's Hospital, Boston, MA, United States; ^15^Partners in Health, Boston, MA, United States; ^16^Division of Infectious Diseases and Vaccinology, School of Public Health, University of California, Berkeley, Berkeley, CA, United States; ^17^MIVEGEC, Université de Montpellier, CNRS, IRD, Montpellier, France; ^18^Faculty of Medicine, University of Antananarivo, Antananarivo, Madagascar

**Keywords:** pandemic response, public health system, health system strengthening, data platform, COVID-19

## Abstract

There are many outstanding questions about how to control the global COVID-19 pandemic. The information void has been especially stark in the World Health Organization Africa Region, which has low per capita reported cases, low testing rates, low access to therapeutic drugs, and has the longest wait for vaccines. As with all disease, the central challenge in responding to COVID-19 is that it requires integrating complex health systems that incorporate prevention, testing, front line health care, and reliable data to inform policies and their implementation within a relevant timeframe. It requires that the population can rely on the health system, and decision-makers can rely on the data. To understand the process and challenges of such an integrated response in an under-resourced rural African setting, we present the COVID-19 strategy in Ifanadiana District, where a partnership between Malagasy Ministry of Public Health (MoPH) and non-governmental organizations integrates prevention, diagnosis, surveillance, and treatment, in the context of a model health system. These efforts touch every level of the health system in the district—community, primary care centers, hospital—including the establishment of the only RT-PCR lab for SARS-CoV-2 testing outside of the capital. Starting in March of 2021, a second wave of COVID-19 occurred in Madagascar, but there remain fewer cases in Ifanadiana than for many other diseases (e.g., malaria). At the Ifanadiana District Hospital, there have been two deaths that are officially attributed to COVID-19. Here, we describe the main components and challenges of this integrated response, the broad epidemiological contours of the epidemic, and how complex data sources can be developed to address many questions of COVID-19 science. Because of data limitations, it still remains unclear how this epidemic will affect rural areas of Madagascar and other developing countries where health system utilization is relatively low and there is limited capacity to diagnose and treat COVID-19 patients. Widespread population based seroprevalence studies are being implemented in Ifanadiana to inform the COVID-19 response strategy as health systems must simultaneously manage perennial and endemic disease threats.

## Introduction

**Figure 1:**
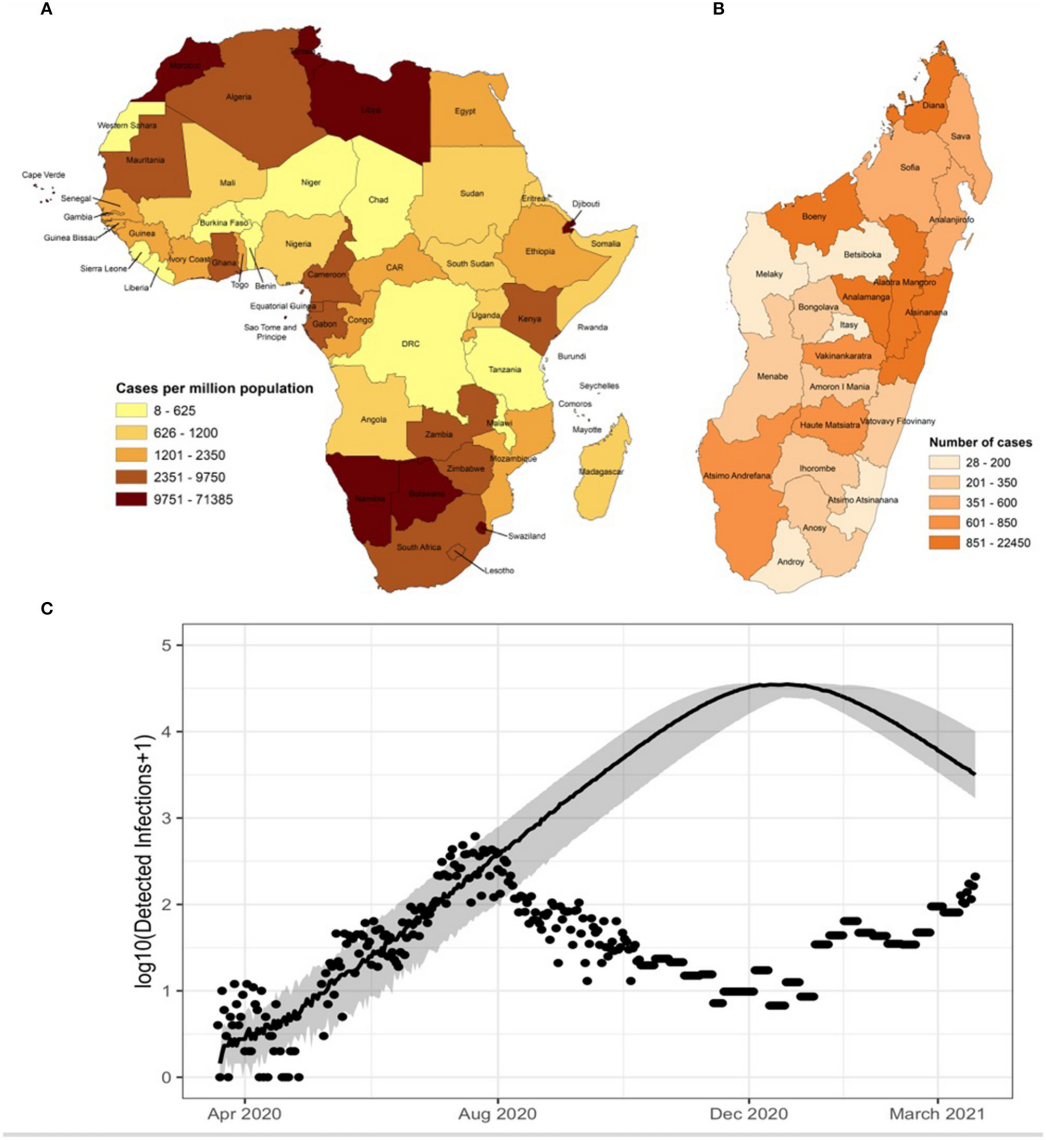
**(A)** Cumulative total cases per million population for each country in the African continent as of April 21 2021 (1). **(B)** Cumulative total cases per region in Madagascar through April 21 2021 (1). **(C)** Updated estimate of COVID-19 dynamics (solid line) based on reported data and mathematical model for Madagascar shows that even conservative models predicted disease prevalence that is orders of magnitude greater than public case reports (dots) to date [for details on this model, see (3)].

As of May 2021, COVID-19 has infected over 160 million people globally and killed more than 3 million, with the highest reported per capita tolls in Europe, North America, and South America in the first year, with the worst surge of the second year occurring in India (1). One of the great mysteries of the pandemic has been its relatively smaller impact on the World Health Organization (WHO) African Region, which endures high burdens of other infectious diseases, including respiratory infections, for reasons that are also relevant to COVID-19 epidemiology: crowding inside and outside of the home, inadequate water and sanitation infrastructure, and under-resourced health systems (2, 3). While Sub-Saharan Africa (SSA) is home to 16% of the world's population, its share of reported COVID-19 cases is 3% of the global case count, more than a year after the first COVID-19 cases were diagnosed in China (Figure 1A) (1). This could be explained by underlying differences in the effectiveness and timing of control measures, immunology, age distribution, contact structure, or low rates of testing and under-reporting (2, 3).

As the world works to roll out newly developed vaccines, the global health community and national governments lack data to guide action throughout most of Africa. This brings to the fore critical questions. Are vaccines especially needed in SSA because of high undetected burdens of COVID-19 that will go untreated? Or is COVID-19 less of a threat in SSA than in the rest of the world while the pandemic response disrupts delivery of health services for other diseases? The central challenge for answering this question is the same for global health equity in the management of most infectious diseases: to ensure that health systems integrate prevention, testing, and treatment of illness for everyone, with quality data that informs the response within a timeframe that matters. This is particularly challenging and important for remote communities, where the majority of the Madagascar population and most of Africa lives (4).

Here, we present the first year of a COVID-19 management and research strategy in the context of a model health district in Madagascar. Key features are the integration of programs across all levels of the local health system (community health, primary care health centers, district hospital), population based preventative efforts, and the array of testing and surveillance approaches necessary for responding to the complex situation. We share lessons learned, identify unresolved questions and show how such a platform can address such critical questions in an under-resourced rural African setting.

Madagascar is a large island nation across the Mozambique channel from mainland Africa, with a population of over 25 million. While its insularity confers advantages to controlling transmission across borders. Madagascar embodies much of the COVID-19 paradox of the rest of SSA: it is managing high burdens of other diseases, including the largest measles and plague epidemics of the past half century (5, 6), while reporting relatively low rates of COVID-19 infection per capita—ranked 27 out of 47 countries in the region (1) (Figure 1A). The first case was identified in the country's capital, Antananarivo, among travelers on 19 March 2020. Community spread was found a week later. Over the course of the first 4 months of the epidemic, cases were identified in localized clusters in three cities in three different regions: Antananarivo, in the central region of Analamanga; Toamasina, on the eastern coast in the region of Atsinanana, and Fianarantsoa, in the southern central region of Haute-Matsiatra. The disease then spread slowly throughout the country. By September 2020, all 22 regions of Madagascar reported active COVID-19 infections (ref). With low testing rates, the true dynamics of the disease are unknown, but there have been several efforts to model its spread based on available data (3, 7, 8). As of April 2021, Madagascar began experiencing a second documented wave of rising cases, which cumulatively total over 30,000 (~1,000 cases per million population) and 520 deaths (18 deaths per million population) recorded in Madagascar (Figures 1B,C). Over 160,000 tests have been administered (~6,000 per million population) with an average positivity rate of 18.75%. The crude case fatality rate of 1.7% is similar to global the average of 2.1%.

Strong health systems are essential for managing disease outbreaks, a lesson reaffirmed during the Haiti Cholera Outbreak in 2010 (9, 10), Ebola crisis in West Africa in 2014–2015 (11, 12), the Middle East Respiratory Syndrome experience in South Korea in 2015 (13, 14), as well as the COVID-19 pandemic in South Korea, Vietnam, Spain and Italy (15–19). To strengthen the local health system, the non-governmental organization, PIVOT, has partnered with the Government of Madagascar to establish a model system for universal health coverage (UHC) in Ifanadiana District (20). Ifanadiana is a rural district of ~200,000 people in the Vatovavy-Fitovinany region in the southeast of Madagascar. This model has several pillars at all levels of the district health system (community health, primary care facilities, and the district hospital): improved system readiness (e.g., infrastructure, staffing, supplies, removal of financial barriers), clinical programs (e.g., infectious disease, maternal, and child health), and quality information systems. The result has been substantial increases in utilization rates of health facilities and community health, as well-progress in neonatal, infant and under-five mortality, and population-based improvements in the coverage of essential services such as antenatal care, delivery in health facilities, and vaccines. For more details on the MoPH-PIVOT model, history, and impact analyses, see (20, 21).

## Madagascar's National Response

**Figure 2:**
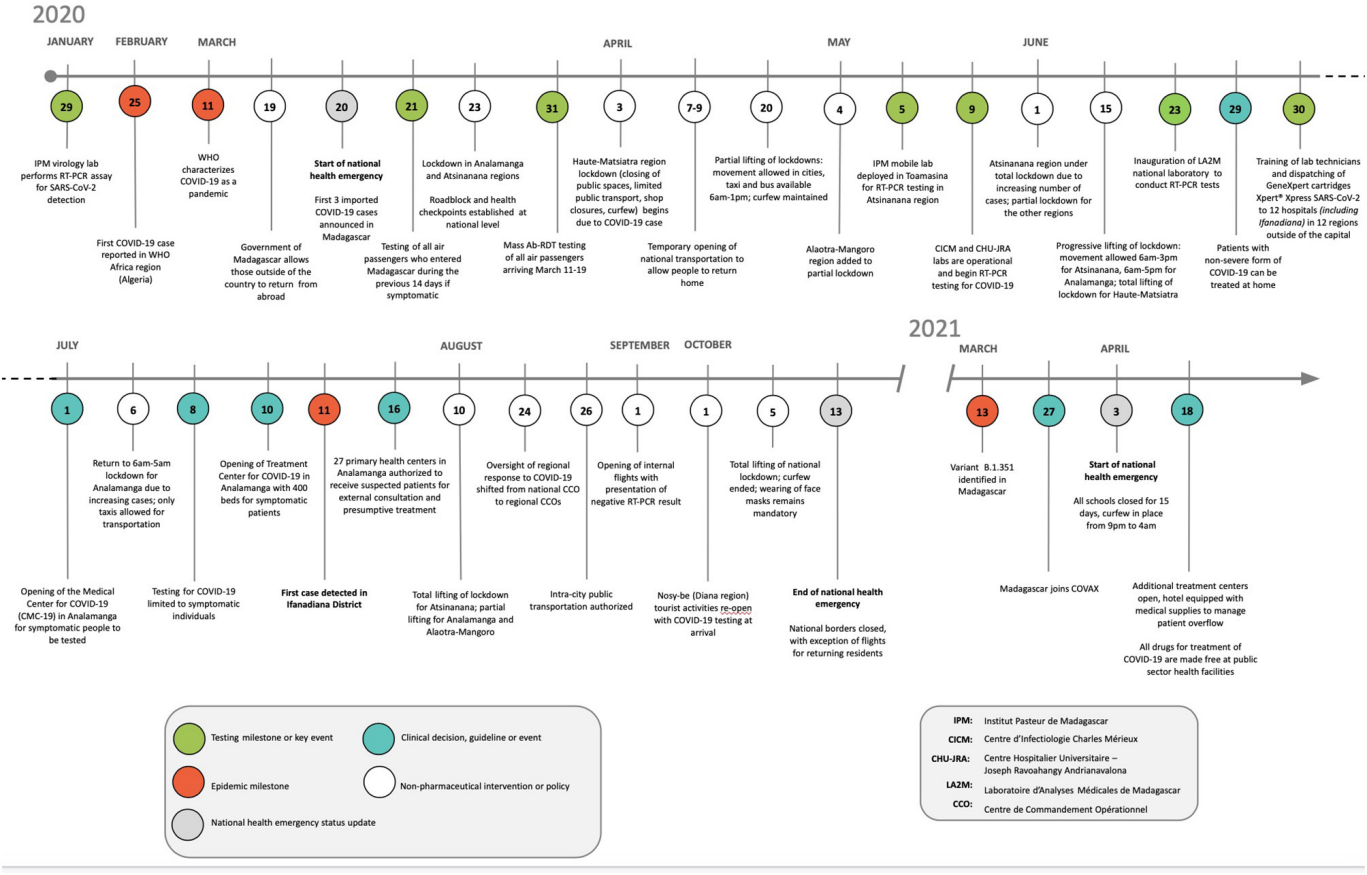
Timeline of policies and interventions in response to COVID-19 in Madagascar.

The Government of Madagascar implemented non-pharmaceutical interventions (NPIs) nation-wide when the first cases of COVID-19 were detected (3). These included barrier measures (mandatory use of facemasks, hand-washing, and social distancing); bans on public gatherings; school closings; and lockdowns in major cities. The international airport was closed to commercial flights, beginning March 20 and continuing until the end of October 2020, and then closed again in April of 2021. Roadblocks were established on all national roads leaving the capital to prevent movement across regional boundaries and public transportation was suspended. NPI implementation and timing presented high variability for each region of the island based on case counts. By early September 2020, as reported cases dwindled and fears of an over-run health system diminished, many NPIs were lifted including restrictions on movement, though loosely enforced mask mandates remained in effect (Figure 2).

Testing is central to disease control, surveillance, and case management. Early in the epidemic, the Government of Madagascar focused testing on screening for possible imported cases. All passengers on international flights during the 2 weeks before the border closure were placed in quarantine for 14 days, after which they were tested for COVID-19. High priority was also placed on contact tracing for initial imported cases, as well as testing of patients with serious, unexplained respiratory illness. When COVID-19 was first introduced in Madagascar, there was one laboratory able to perform molecular testing for SARS-CoV-2. To meet the need for expanded testing, four molecular labs in the capital were made available for testing SARS-CoV-2 via RT-PCR. These relied on the transport of nasopharyngeal swabs of suspected cases from health facilities around the country to Antananarivo. In addition, over time 12 district and regional hospitals that were already equipped with GeneXpert machines (routinely used for TB diagnosis) were supplied with specific SARS-CoV-2 cartridges in order to decentralize testing.

At the start of the epidemic, all Government of Madagascar COVID-19 response activities were managed by a national central-level Operations Coordinating Center (CCO in French). As the epidemic progressed, management authority shifted to regional CCOs. At the district level, a Vigilance Committee, including local authorities and partners, was established to manage COVID-19 response.

## Ifanadiana District Response

The first confirmed case of COVID-19 in Ifanadiana District was reported on July, 11 2020. As of May 2021, there have been 136 officially reported cases of COVID-19 in the district, including 12 severe cases and 2 official deaths. Administrative districts in Madagascar are similar to districts in much of Africa. As with many health districts in Africa, it has one district hospital. The district is divided into 15 communes (local administrative municipalities, each with at least one primary health center), which are further subdivided into fokontany—a cluster of villages, with a population of ~300–4,400 individuals. Each fokontany has a community health site where community health workers provide treatment for sick children under-five, family planning counseling, and screening for tuberculosis (TB) and malnutrition in some locations.

As in other rural areas in the region, Ifanadiana District has limited water and sanitation infrastructure, necessary to reduce disease transmission. Approximately 15% of the population has access to clean water and <3% have access to a toilet or improved latrine (22). Under-five mortality, which is 89 per 1,000, is driven largely by malaria, diarrhea, and respiratory infections (19). The burden of TB in Madagascar (incidence of 233 per 100,000) is similar to many mainland African countries (23). The determinants of other infectious diseases, including the large and multigenerational structure of households (22, 24), are potential drivers of COVID-19 transmission in the community.

The response to COVID-19 in Ifanadiana District focused on: (1) preparing the community and delaying disease introduction; (2) bolstering the health system response through material support, direct clinical care, and support of patients; (3) expanding testing and lab capacity; and (4) information systems, surveillance, and modeling. Preparations were made at all levels of the health system (community, basic health center, and hospital) (Table 1).

**Table 1:** COVID-19 interventions across the levels of the health system in Ifanadiana District.

	**Community**	**Health center**	**District hospital**
Prevention	•Mask distribution •Door-to-door education campaign on COVID-19 risks and prevention •Community health posts equipped with buckets of water and soap to promote hand hygiene	•Pre-triage screening of all patients presenting for care •Construction of well-ventilated pre-triage areas •Video and education activities on COVID-19 risks and prevention	•Pre-triage screening of all patients presenting for care •Construction of well-ventilated pre-triage areas •Video and education activities on COVID-19 risks and prevention
Diagnosis	•Community health workers refer children under five diagnosed with fever or other COVID-19 symptom but negative malaria RDT to health center	•COVID-19 Ag-RDTs •Transfer of nasopharyngeal swab samples to district hospital or RT-PCR lab for NAAT	•COVID-19 Ag-RDTs •NAAT confirmation testing with Xpert or transfer of samples to RT-PCR lab
Treatment		•Symptom management •Isolation recommended for confirmed cases	•Symptom management •Confirmed cases with severe symptoms provided inpatient care on COVID-19 ward with enhanced ventilation and available oxygen •Isolation recommended for confirmed cases
Research activities		•Antibody serosurveillance in general patient population •Antigen and antibody testing of healthcare workers	•Antibody serosurveillance in general patient population •Antigen and antibody testing of healthcare workers

### Preparing the Community and Delaying Disease Introduction

**Figure 3:**
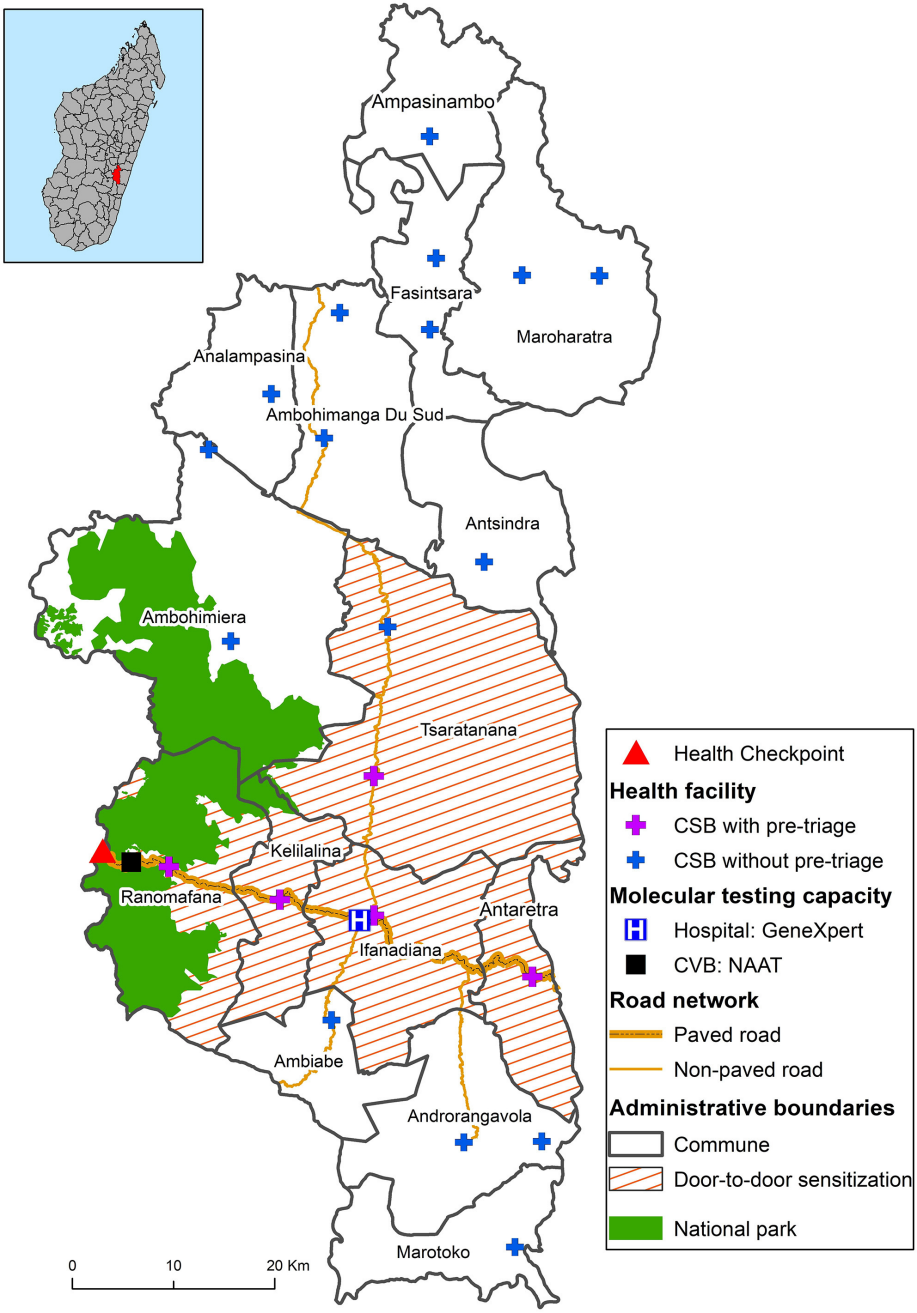
Map of the COVID-19 response in Ifanadiana District with interventions, clinical care, testing capacity, and research activities across levels of the health system.

District-wide COVID-19 preparedness activities began with multisectoral planning committees before the disease was first diagnosed in the district. Nationally and within Ifanadiana District, the initial government response focused on slowing the spread of COVID-19 throughout Madagascar by putting in place restrictions on movement and on screening travelers moving between towns and regions. The Government of Madagascar closed national roads to public transportation and established health checkpoints to screen for COVID-19 symptoms. Local partners, led by the gendarmerie (in charge of law enforcement), supported the health checkpoint established on National Road 25 (Figure 3); all passengers arriving by motor vehicle passed through this screening checkpoint before entering the district. From April 2 to August 9, all travelers along national road 25 were required to stop for a temperature check. Individuals with a temperature above 38°C (100.4°F) were questioned about their health status and their city of origin, in order to identify the risk of possible infection.

Several NPIs, including use of face masks, hand-washing, social distancing, and other barrier measures were implemented in the district to prevent and mitigate disease transmission. Community sensitization was carried out via radio and special informational programs broadcast by local radio stations, posters, and videos at primary care health centers and the district hospital. Additionally, sensitization campaigns were carried out in five communes via community education sessions and door-to-door visits by health promotion staff (Figure 3). Education sessions on COVID-19 prevention and detection of symptoms were conducted on market days and at schools. Approximately 40,000 facemasks were donated and distributed to the community.

### COVID-19 Clinical Care

Comprehensive guidelines were developed for COVID-19 prevention, transmission, diagnosis, and management. The guidelines, available in French and English, were based on guidance from the WHO (25, 26), the US Centers for Disease Control and Prevention (27) and the international NGO, Partners In Health, and adapted for the local context of Ifanadiana District (16). They included basic information about the SARS-CoV-2 virus; reorganization of healthcare facilities for improved infection prevention and control; diagnostics and clinical care; data collection; and supply chain systems. Guidelines were made available to clinical staff at the district and national level. Prevention, diagnosis, and treatment measures were implemented across all levels of the health system.

At both health centers and the district hospital, pre-triage sites were established in well-ventilated spaces (such as gazebos) to screen for suspected COVID-19 cases and to prevent close congregation indoors. During pre-triage, each patient had their temperature taken and was asked about COVID-19 symptoms and about any potential recent exposures. Patients who screened negative for symptoms and exposure were directed to the service that they sought. Suspected cases and those who screened positive for COVID-19 exposure were directed to a specific area where a dedicated health care worker in personal protective equipment (PPE) evaluated them for COVID-19. If, after further evaluation, testing was deemed appropriate, a nasopharyngeal sample was collected for testing either by GeneXpert or rapid antigen test (Ag-RDT) (described below). Implementation of testing varied across the District with the majority of tests deployed at health facilities along the road. However, as of May, 2021, rapid antigen testing has been deployed to health facilities across the District.

Patients who tested positive for COVID-19 were advised to isolate. An isolation facility was set up away from the hospital grounds for people with mild COVID-19 who did not require hospitalization, but who could not safely isolate at home. Patients with severe symptoms were admitted for inpatient care at the district hospital. Patients with minor symptoms and asymptomatic cases were advised about disease progression, the need for isolation, and danger signs that would require a return to the health facility. Confirmed cases were provided with psychological support from PIVOT's social workers if they requested it.

Whenever possible, close contacts of confirmed cases were tested for COVID-19, instructed to quarantine, and followed for 14 days while monitoring for symptom development; contacts were tested if COVID-19 symptoms developed. To support individuals undergoing quarantine, food and toiletries were provided whenever possible.

The district hospital was reorganized to prepare for a surge of severely ill patients who might require isolation and/or oxygen. One section of the hospital—which had already been designated as an Infectious Disease Ward, and which is located away from the rest of the hospital wards and officers—was dedicated to receive severe COVID-19 patients and another tent-based structure nearby was prepared for patient overflow. Suspected cases were kept separate from confirmed cases. Procedures were put in place to minimize transmission from healthcare workers to patients: providers rounded on either COVID-19 patients or non-COVID patients, or saw patients without COVID-19 first, and then attended to patients with COVID-19 while in full PPE.

Critical materials were obtained to meet the needs of patients with respiratory distress. These included additional oxygen concentrators, canisters, tubing, and masks, as well as pulse oximeters, blood pressure cuffs, vital sign monitors, ancillary medications (such as dexamethasone, and IV fluids. Plans were also made for possible delays in supply chain systems for critical items unrelated to COVID-19, such as malaria rapid diagnostic tests (RDTs) and medications. To reduce transmission by and to health care workers, PPE, including surgical masks, N95 masks, gloves, goggles, disposable gowns and foot-covers, were provided to the district health office, and then distributed to health facilities (19, 21, 22).

### Increasing COVID-19 Testing Capacity in Ifanadiana District

One of the most fundamental steps to respond to COVID-19 is reliable testing data for patient diagnostics, planning, and research. The testing landscape is exceedingly complex and evolving in real time due to changes in technologies, their validation, clinical capacity and local and global supply chains. According to government policy, suspected cases are confirmed by a gold standard nucleic acid amplification test (NAAT)— RT-PCR or GeneXpert— to be included in the national COVID-19 statistics. Since the start of the pandemic, Madagascar has completed over 200,000 tests. There are four laboratories performing confirmation tests by RT-PCR, all of which are located in the capital city of Antananarivo.

Rapid technological advances led to several COVID-19 testing options, including the Xpert® Xpress SARS-CoV-2 cartridge, which relies on existing GeneXpert machines. This enabled rapid deployment of a test that can generate results within 45 min. As the disease spread to all 22 regions of the country, the Malagasy MoPH deployed thousands of Xpert® Xpress SARS-CoV-2 cartridges to twelve hospitals around Madagascar (including Ifanadiana District Hospital) that are equipped with this instrument in order to decentralize COVID-19 diagnostics. Under current national guidelines, diagnosis by GeneXpert is limited to suspected cases, although testing criteria continue to evolve. The MoPH defines suspected cases as (1) all people presenting with cough, sore throat, or dyspnea, with or without fever and having been in close contact with a confirmed case, (2) all people or clusters of people suffering for severe acute respiratory illness and having been in contact with a confirmed case. Though protocols were established by the MoPH for contact-tracing, they have not been implemented routinely throughout the country.

GeneXpert alone cannot adequately meet diagnostic demand in Madagascar due to insufficient availability of cartridges. In addition, there are limitations in the transportation of samples and communication of results. In order to improve COVID-19 diagnostic capacity, PIVOT and the MoPH implemented one of the only programs for COVID-19 antigen rapid diagnostic tests (Ag-RDTs) in the country, using the SD BIOSENSOR® STANDARD Q COVID-19. This point-of-care test can be administered provide results within 15 min, and does not require laboratory training for test administrators. It has good clinical performance, with sensitivity and specificity ranging from 58–89%, and from 92–99%, respectively (28–30); sensitivity is higher during the first 5 days after the symptom onset (28, 29). The Ag-RDTs were donated to the District Health Office, and distributed to various health centers and the district hospital (Figure 3).

In addition, the Ministry of Public Health, PIVOT, and Centre ValBio (CVB) partnered to increase RT-PCR capacity for COVID-19 NAAT outside of the capital. CVB is a conservation, education, and research organization located along the national highway near Ranomafana National Park in Ifanadiana District, with a biosafety level 2 laboratory. An RT-PCR machine was procured to allow for middle to high throughput molecular confirmation of COVID-19. The lab has been outfitted with needed equipment, reagents, and staff trained in accordance with MoPH protocols. The lab, launched in May 2021, following international procurement challenges, increases testing capacity not only for Ifanadiana District, but also for the region, extending access to vital diagnostic services for the rural population. It will also reduce the turnaround time for PCR test results, allowing for better management and mitigation of the spread of COVID-19.

### COVID-19 Research Platform

What are the optimal strategies for COVID-19 control and how should that change over time? More than a year after the first reported case of COVID-19 in Wuhan, China, the answer to these questions remain unclear for many low and middle-income countries including Madagascar. Answers require accounting for epidemiological, molecular, and global health delivery considerations, and they vary across populations and social/political/epidemiological context. We highlight a few of the highest priority questions necessary to determine optimal control strategies (Box 1).

Box 1 **Research questions.**

Epidemiological

a) What is the burden of COVID-19 compared to other diseases and how is that changing over time?

b) What are the basic parameters of the disease: *R*_0_, clearance, loss of immunity, fatality?

c) What are the primary risk factors for viral transmission, morbidity, and mortality?

Biological

a) What explains heterogeneity in immunological responses and clinical manifestations?

b) What is the duration of acquired immunity?

c) How does viral evolution affect transmission and disease, and how is fit influenced by acquired or vaccine-driven immune response?

Delivery/Implementation

a) What are the direct and indirect costs of various control measures?

b) What are their impacts on other dimensions of the health system?

c) Vaccines: for whom, where, which, how and how often?

Socio-economic

a) What individual and community-level factors are associated with infection and diagnosis?

b) How does geographic inequity impact health seeking for COVID-19 in a rural, mountainous district?

c) What is the impact of the disease on economic, social, emotional, and physical well-being?

**Figure 4:**
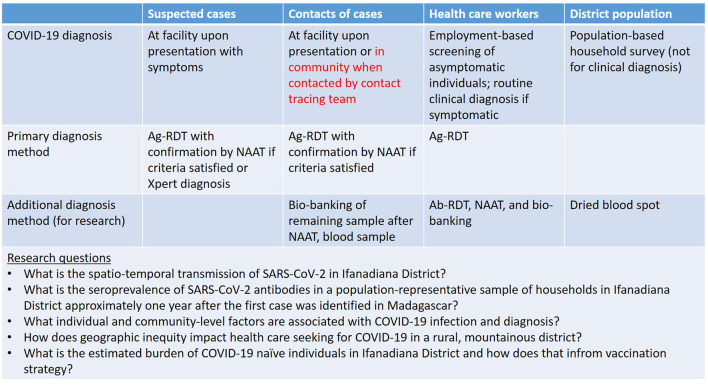
Key populations for COVID-19 research activities and data collection to inform modeling.

Since 2014, PIVOT and the MoPH have partnered to pioneer new ways of integrating field-based data analytics to improve health services. This combination of health systems and science presents unique opportunities for addressing the COVID-19 information crisis. For COVID-19, we collect data from a range of sources—no single one of which adequately captures the disease dynamics that are changing over space and time (Figure 4). Broadly, the main classes of quantitative data are from: (1) patients within the health system (e.g., health management information systems and patient diagnoses); (2) general population outside of the health system (e.g., household surveys that include biomarkers); and (3) the environment (e.g., environmental sampling and geographic information systems). These quantitative data are triangulated with other information sources: anecdotal reporting and programmatic updates from front line health workers, quantitative data on other indicators of the health system (availability of tests, health system utilization, geography, stock outs, and dynamics of other infections/health services), and analytical methods including mathematical modeling that are all material for assessing COVID-19 epidemiology. Such methods of combining complex data sources at different spatial and temporal scales for projecting dynamics of malaria (“nowcasting”) have been recently employed for Ifanadiana District (31).

One of the most important initiatives is a seroprevalence study, initiated in April 2021, which has been integrated into a pre-existing longitudinal cohort study designed to track general population health conditions (mortality rates, vaccine uptake, and access to care). The cohort consists of a representative sample of 1,600 households (~9,000 people) in 80 geographic clusters across Ifanadiana District, for whom we currently have data from 2014, 2016, and 2018 (24). Questionnaires are based on the internationally-validated Demographic and Health Surveys, and include information on household composition, indicators of socio-economic status, recent illness for all household members, women's reproductive history, and mortality, as well as preventive and curative care seeking. The first three waves of the cohort study (2014–2018) included biometric measurements of all household members. In 2021, dried blood spot (DBS) tests were added to support a range of serological tests, including for SARS-COV-2 antibodies to be analyzed in partnership with the Pasteur Institute of Madagascar.

Point estimates of seroprevalence a year after introduction of the virus will provide a strong indication of the attack rate of COVID-19 in rural Madagascar. When combined with spatiotemporal data from patient diagnostics and mathematical modeling, this can be directly used for estimating effects of disease control strategies (i.e., vaccines) over time.

## Conclusion

More than a year after the first case of COVID-19 was diagnosed in Wuhan, China, there remain enormous challenges for disease control globally, and unanswered questions that threaten strategies for prevention efforts, compromise strategic health system priority-setting, and undermine pandemic preparedness for the future. A unifying concept of global health is the “know-do gap”; known solutions are often not implemented because of break downs in the functioning of the health system itself. The system for responding to COVID-19 is the same as that for managing other diseases. It must be integrated, strong, and data-driven to be able to adapt dynamically to operational and epidemiological changes. But as a novel pathogen, COVID-19 also presents unique challenges due to acute information gaps that greatly confound disease control.

What explains the enormous heterogeneity in disease outcomes within countries and around the world? Biological factors, such as age and pre-existing conditions are clearly important, as are social determinants such as race and income. However, the impacts of COVID-19 on low-income countries, particularly those in the WHO African Region, are reportedly lower than high-income countries. This paradox suggests fundamentally different epidemiology, management, or rates of testing and reporting, with enormous implications for managing COVID-19 and preparing for future pandemics. To answer these questions, strong health systems are necessary, but not sufficient.

The need to strengthen health systems, generate reliable data, and make scientific advances to battle disease, are mutually reinforcing goals. In rural Ifanadiana District, COVID-19 management focused on integrated community preparedness, improvements to clinical infrastructure and processes, infection prevention, increased testing capacity, clinical care, and support for patients. These activities were designed to both ensure quality care for patients and reduce viral transmission. In responding to the pandemic, Madagascar drew from its experience with past national epidemics and from global recommendations for COVID-19 mitigation, which included early and widespread adoption of NPIs that were later relaxed before being enforced again during the second wave in March, April and May of 2021.

But there have also been many challenges to fully implement an emergency response, and to transition that response to a sustained level of vigilance. Due to a range of factors—including supply chain challenges, health system fatigue, economic constraints, sensitization and trust, and the complexity of evolving politics and policy—patients are still likely to be under-screened, under-tested, and cases are likely under-reported. Health workers, decision-makers, and communities, became fatigued from extensive and costly preparation for the early threat of the pandemic, leaving the health system exposed to the second major wave that started in March, 2021. PPE is under-resourced and health workers have become especially vulnerable in the second wave. The lack of adequate resources for isolation, accompaniment, and treatment of positive cases undermine incentives for logistically complicated screening and testing.

Our understanding of the effects of these efforts even on local areas like Ifanadiana District are not yet clear. Anecdotal evidence from front line health workers, combined with existing health system data, show high burdens of other infectious diseases such as malaria, pneumonia, and diarrheal disease, than of COVID-19. But suspected cases are not consistently tested. Two key sources of data continue to be critical moving forward: patient-based diagnostics and population-based serology. GeneXpert, RT-PCR, and validated Ag-RDTs are increasingly being relied on to ensure systems for tracking dynamics of disease but require sourcing that is reliable, protocols that are well-established and enforced, and patient buy-in. When complemented by seroprevalence studies on a representative sample of the population, our understanding of the current burden of disease will clarify quickly. Advances in modern serology and next generation sequencing, when combined with such integrated platforms, are within reach and could revolutionize disease surveillance to prevent the next pandemic and are within reach. Yet, there remains inadequate funding, forcing countries like Madagascar to face sharp tradeoffs for priority-setting for other diseases. International interest in transparent data and surveillance can conflict with domestic policy objectives and national sovereignty. Our platform shows the potential of having built-in epidemiological and health data systems, and that such efforts can be done in combination with government and non-governmental partnership, but these efforts also reveal complex challenges even in the best of circumstances.

As with Ebola, plague, measles, and other recent epidemics, COVID-19 has revealed fundamental weaknesses in existing health systems. The Malagasy public health system, like those throughout much of the world, can learn from the COVID-19 experience to be better prepared to confront future epidemics. We show how an integrated platform of strengthened health systems and research can be established in order to understand and control the pandemic.

## Data Availability Statement

Publicly available datasets were analyzed in this study. This data can be found here: Data for Madagascar can be accessed through Madagascar Ministry of Public Health. Data were also obtained at: https://www.worldometers.info/coronavirus/.

## Author Contributions

The clinical intervention was designed by RR, HA, BRaz, ER-F, VR, FR, AT, LRah, LRak, JH, LC, GC, TL, MR, ARan, ER, GR, and AMa with laboratory and testing interventions designed by RR, LRah, LRak, LC, MK, PW, TG, MD, AMa, KF, and MB. HA, BRaz, ER-F, GR, JR, KF, and MB performed data collection and drafted the article. RR, HA, BRaz, ER-F, ME, FI, AG, AMa, KF, and MB conducted data analysis and interpretation. All authors contributed to the conception of the study initiative and critically reviewed the manuscript and approved the final version to be published.

## Conflict of Interest

The authors declare that the research was conducted in the absence of any commercial or financial relationships that could be construed as a potential conflict of interest. The reviewer SN declared a shared affiliation, with no collaboration, with one of the authors AG, to the handling editor at the time of the review.
